# ﻿*Licariaramiroi* (Lauraceae), a new species from Western Mexico

**DOI:** 10.3897/phytokeys.218.94356

**Published:** 2023-01-10

**Authors:** Ramón Cuevas Guzmán, Enrique V. Sánchez-Rodríguez, José Guadalupe Morales-Arias

**Affiliations:** 1 Laboratorio de Botánica, Departamento de Ecología y Recursos Naturales, Centro Universitario de la Costa Sur, Universidad de Guadalajara, Av. Independencia Nacional 151, 48900 Autlán de Navarro, Jalisco, Mexico Universidad de Guadalajara Autlán de Navarro Mexico

**Keywords:** cloud forest, endemism, laurel forest, Neotropic, new species, tropical subdeciduous forest

## Abstract

*Licariaramiroi*, a species endemic to western Mexico, is described and illustrated. The ascription of the new species to Licaria is analysed. It is classified in the subgen. Licaria and is most closely related to *L.triandra* and *L.siphonantha* from which it differs by its glabrous vegetative and floral structures, stamens mainly with free anthers and the inner wall of the receptacle sericeous. According to the IUCN criteria, the species is classified as critically endangered.

## ﻿Introduction

Lauraceae Juss. are a family that is poorly known in terms of the number of taxa and distribution. This is attributed to the fact that many species are large trees with small and inconspicuous flowers, which are difficult to locate and collect (van der Werff and Richter 1996), although these structures are indispensable for the identification of genera and species in the family (van der Werff and Richter 1996; Lorea-Hernández 2002). The family comprises 2,500–3,000 species and is best represented in the tropics of America and Asia (van der Werff and Richter 1996; van der Werff 2009b). The Lauraceae include 120 species in southern Mexico, which are considered to represent between 90 and 93% of the Mexican Lauraceae (Lorea-Hernández 2002).

*Licaria* Aubl. is a Neotropical genus of around 60 species, of which at least 20 have been described in the last 25 years (van der Werff 2009a). Of the total number of *Licaria* species, 17 have been recorded for southern Mexico, eight of which are considered endemic to the country (Lorea-Hernández 2002). Species of *Licaria* are easily recognised by the presence of fertile stamens in whorl III only, bisporangiate anthers and fruits on a well-developed cupule, usually with a double margin (Burger and van der Werff 1990; van der Werff 1991; Lorea-Hernández 1999; Kurz 2000; van der Werff 2009a). The subgenera *Armeniaca*, *Canella* and *Licaria* are recognised by the anther shape and direction of opening and the presence or absence of staminodes in series I and II, with the latter of the three subgen. presenting the greatest species richness and distribution ranges from Florida on the Atlantic side and Mexico (Sinaloa) on the Pacific slope, to southern Brazil and Bolivia (Burger and van der Werff 1990; Kurz 2000; van der Werff 2009a).

As part of a gradient analysis study of the vegetation of the El Tecolote canyon in the Sierra de Manantlán, Jalisco, Mexico (Cuevas 2002), material was collected from a *Licaria* that could not be identified. Initially, only sterile material or material with fruits was available, then material with flowers was collected. However, given the characteristics observed in the flower, particularly the variation in the number of stamens, it was decided to wait until more collections with flowers became available. After making detailed studies of the material from the Sierra de Manantlán, we found no accommodation amongst the known species of *Licaria*, for that reason, this is proposed as a new species.

## ﻿Materials and methods

Species morphology was studied and described, based on specimens available in the ZEA Herbarium of the University of Guadalajara and on fresh material collected in the field. Vegetative and reproductive structures were carefully revised under a stereoscopic microscope using the protocol recommended by Radford et al. (1974) in order to elaborate the description. Relevant literature was reviewed, including taxonomic treatments of the genus *Licaria* and descriptions of new species in the genus from the last 25 years (Burger and van der Werff 1990; van der Werff 1991; Gómez-Laurito and Cascante 1999; Lorea-Hernández 1999; Kurz 2000; van der Werff and Vicentini 2000; Lorea-Hernández 2005; van der Werff 2009a). Photographs of type specimens of species that could be related to the *Licaria* species, proposed here as new, were reviewed in the Tropicos (2022) (www.tropicos.org) and JSTOR (2020) (http://plants.jstor.org) databases. Measurements and photographs of the floral structures were taken using fresh or rehydrated material; all other structures were measured in dehydrated specimens. Microphotographs were taken with a SteREO Zeiss Discovery V12 stereoscopic microscope connected to an AxioCam 305 Color Zeiss camera and Zen 3.2 (blue edition) software. Photographs of the species in the field were taken with a Canon EOS M3 camera. Habitat and phenology data of the new species were obtained from herbarium specimen labels and fieldwork, while information pertaining to the related species was taken from the relevant literature (Kurz 2000; Lorea-Hernández 2005).

## ﻿Results

**Figure 1.**
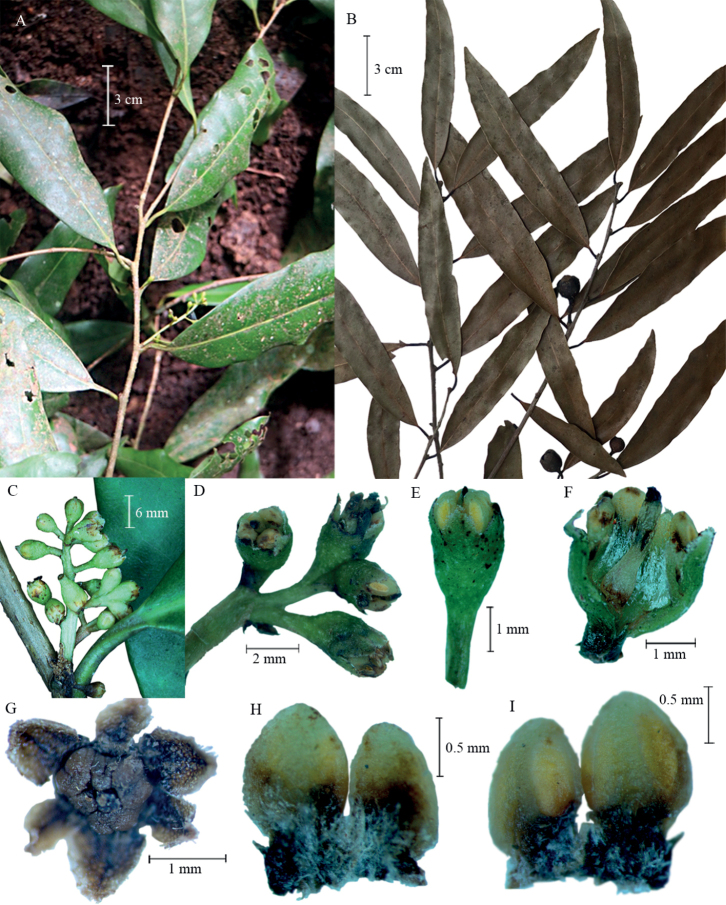
*Licariaramiroi* Cuevas sp. nov. **A** branchlets with inflorescences **B** branchlets with immature fruit **C** close-up image of an inflorescence **D** inflorescence with flowers in top and side view, with the two terminal flowers fused at their bases **E** side view of flower **F** flower in tangential section showing pistil and sericeous internal surface of hypanthium **G** upper frontal view of a flower with stamens the tepals opened artificially **H** Pair of stamens in adaxial view **I** Pair of stamens in abaxial view.

The morphology characteristics of the plant material, collected in the El Tecolote Canyon, supports its allocation in the genus *Licaria* (Fig. 1). All the plants analysed have flowers with fertile stamens only of the whorl III, bisporangiate, lack of staminodes in the first two whorls and the fourth, a deep receptacle surrounding the whole ovary and fruits with double-rimmed cupule. At the same time, the combination found in the specimens of narrow, glabrous leaves, simple cymose-paniculate, glabrous inflorescences, glabrous tepals, stamens fused just by the base of the filaments and receptacle sericeous inside, do not correspond to any *Licaria* species known in Mexico or elsewhere (Table 1); therefore, the recognition of a new species is presented here.

**Table 1.** Morphological differences amongst *Licaria* species and some additional information regarding their phenology and distribution. Information about the species was obtained from Burger & van der Werff (1990) and Kurz (2000) for *L.triandra* and Lorea-Hernández (2005) for *L.siphonantha* and the present study for *L.ramiroi*.

Character	*L.ramiroi*	*L.triandra*	*L.siphonantha*
Leaf blade shape	Lanceolate to oblong	Lanceolate to elliptic	Narrowly elliptical
Leaf blade consistency	Chartaceous	Coriaceous	Chartaceous
Leaf blade surface	Glabrous	Blades when young puberulent on the abaxial part, then glabrous	Glabrous adaxially and appressed puberulent to glabrescent abaxially
Leaf blade size (cm)	7–16 × 1.3–4.7	5–15 × 2–7	(9)13.5–19(-22) × (2-)3.5–5.5
Secondary veins (pairs)	12–23	5–10	12–17
Petiole length (mm)	(4-)7–12 × 1–1.8	8–20 × 1–2	(7–)11–14(-17) × 1–1.8
Inflorescence size (cm)	1–4	1.5–13	3–8(-11.5)
Inflorescence surface	Glabrous, sometimes with few appressed hairs	Sparsely to densely appressed pubescent	Densely puberulent
Pedicel length (mm)	1.2–1.8	1–4	(0.2-)0.7–1.2(-1.5)
Flower length (mm)	2.5–3	1.5–3	2.2–2.5
Receptacle indument	Glabrous outside and sericeous inside	Glabrous to compressed hairy on the outside and occasionally with a few hairs on the inside	Glabrous to appressed puberulent on the outside and glabrous on the inside
Tepal size (mm)	0.8–1.3 × 0.8–0.9	0.7–1	1–1.2 × 0.8–1
Stamens in whorl III	3–5	3	3
Stamen size (mm)	1.2–2.4	1.1–1.7	1.6–1.8
Anther size (mm) and condition	1.1–1.3 × 0.8–0.9, free	Size data unavailable, fused at least for its basal half	1, fused for its basal third
Number and condition of glands	4–8, some free and others fused on their bases	3–6, fused at least for the lower half of their bases, rarely solitary	Number data unavailable, free
Cupule size (mm)	12–14 × 14–18	6–15 × 12–25	8.5–10.5 × 15.5–17.5
Fruit size (mm)	20–28 × 12–15	12–30 × 8–15	16–18 × 11–12.5
Flowering period	July to August	January to December	April to May
Ripe fruit period	February to March	January to December	April to May
Distribution	Jalisco	From Florida, on the Atlantic side and Sinaloa, through the Pacific slope, to Bolivia.	Guerrero

### ﻿Taxonomic treatment

#### ﻿ Licaria ramiroi

Taxon classificationPlantaeLauralesLauraceae

Cuevas sp. nov.

06AA8D8D-E18E-5DAF-B784-3818DBB091A7

urn:lsid:ipni.org:names:77311681-1

Figs 1 , 2

##### Diagnosis.

*Licariaramiroi* is similar to *L.triandra* (Sw.) Kosterm. but differs from the latter by leaves with 12–23 pairs of secondary veins (vs. 5–10); inflorescence surface commonly glabrous (vs. sparsely to densely appressed pubescent); entire hypanthium sericeous inside (vs. glabrous); stamens with free anthers (vs. stamens with anthers fused at least in their basal half).

##### Type.

Mexico. State of Jalisco: Municipality of Casimiro Castillo, cañada El Tecolote, 19 July 2022 (fl), *R. Cuevas G., E.V. Sánchez R. & J.G. Morales A. 14182* (holotype ZEA; isotypes to be distributed).

##### Description.

Trees 10–26 m in height, trunk 15–60 cm in diameter; bark flaky and peeling off in irregular layers, the outer reddish-brown, the inner yellow, turning pink as it oxidises. Branchlets cylindrical, 1.5–2 mm thick, brownish-grey, lenticellate and exfoliating, glabrous; axillary buds conical, 1.5–2 mm in length, 1 mm in width, glabrous; terminal buds cylindrical, 7–12 mm in length, 1 mm in width, reddish, glabrous. Leaves alternate, reddish when sprouting, mature leaves lanceolate to oblong, 7–16 × 1.3–4.7 cm, 3–6 times longer than wide, chartaceous, glabrous on both sides; base cuneate, sometimes obtuse; margins entire, sclerotic; apex acuminate; the secondary veins are inconspicuous, but recognisable on both sides, whereas the higher-order venation is not resolved, mid-vein depressed above and protruding below, secondary veins 12–23 pairs; petioles sulcate adaxially (4–) 7–12 × 1–1.8 mm, glabrous. Inflorescences axillary, paniculate-cymose, branched from near the base, rarely solitary, 1–4 cm in length, the terminal flowers often fused at their bases; peduncles 1–2 mm in length; bracts soon deciduous, concave, ovate-lanceolate, 0.7–1.9 × 0.4–0.8 mm, glabrous. Flowers ellipsoid or obovoid, 2.5–3 mm in length; pedicels 1.2–1.8 mm in length, glabrous; hypanthium 1.4–2.1 × 1.9–2 mm, externally glabrous, internally sericeous; tepals 6, fused to 1/4 of their length in the basal part, imbricate, ovate, subequal (inner ones slightly smaller), 0.8–1.3 × 0.8–0.9 mm, concave, inconspicuously fimbriated on their margins; stamen series I and II lacking; androecium with 3–5 stamens, exserted, fused at the filaments only, tomentose on both sides of filaments, 1.2–2.4 mm in length, anthers bisporangiate, ovate to oblong, tomentose at the base on the adaxial face, the rest glabrous, 1.1–1.3 × 0.8–0.9 mm, sporangia on the abaxial side of the anther, each sporangium with an oblique locule covering most of the anther body, sometimes the locule sagittate or irregular i.e. two locules fused, valves extrorse and remaining at the apex of the anther; 4–8 glands observed, at the base of the filaments or slightly above, sometimes contiguous glands fused at their bases, but with clearly separated apices, lanceolate to ovate or irregularly shaped, lamellar, 0.4–0.6 × 0.2–0.4 mm, pubescent at their base; pistil elongate, 2.3–2.7 mm in length, glabrous, ovary 0.8–1 × 0.6–0.7 mm. Fruit ellipsoid, purple with a yellow base when mature, 20–28 × 12–15 mm, glabrous; cupule lenticellate, 12–14 mm in height, 14–18 mm in diameter at apex, 7–10 mm in depth, double-rimmed, outer margin slightly ascending to spreading, wavy, the inner margin erect, entire, 0.5–1 mm in height; pedicel in fruit turbinate, 3–5 mm in length, 3–4.5 mm in diameter distally, 1.5–2 mm basally.

##### Distribution and ecology.

*Licariaramiroi* is known from only one locality in the Sierra de Manantlán, Jalisco, Mexico. It has been recorded between 1000 and 1600 m in elevation in an ecotone between subdeciduous tropical forest and montane cloud forest. Dominant trees in this habitat correspond to individuals of the Lauraceae family, such as *Beilschmiediamanantlanensis* Cuevas & Cochrane, *Damburneyarudis* (C.K. Allen) Trofimov & Rohwer and *D.salicifolia* (Kunth) Trofimov & Rohwer. Other tree species observed are *Amyrismexicana* Lundell, *Aphananthemonoica* (Hemsl.) J.-F. Leroy, *Calatolalaevigata* Standl., *Cedrelaodorata* L., *Drypetesgentryi* Monach., *Myrcianthesfragrans* (Sw.) McVaugh, *Prunuscortapico* Kerber ex Koehne, *Sideroxylonbrucebenzii* Cuevas & A. Vázquez, *S.portoricense* Urb. and *Trophismexicana* (Liebm.) Bureau. Hemi-epiphytes are represented by *Balmeastormiae* Martínez, *Ficusaurea* Nutt., *Oreopanaxechinops* (Schltdl. & Cham.) Decne. & Planch. and *Oreopanaxsanderianus* Hemsl. and vines by *Solandramaxima* (Sessé & Moc.) P.S. Green.

Field observations were carried out for three years; as we found no flowers or fruits during the different seasons of the year, nor observed any seedlings, in this period, this leads us to suggest that this species could have supra-annual flowering and fruiting periods.

The locality from where the new species of *Licaria* is described is an area that has been prominent for records of new species, such as *Beilschmiediamanantlanensis* (Cuevas & Cochrane, 1999), *Sideroxylonbrucebenzii* (Cuevas & Vázquez-García, 2021) or disjunct distributions, such as those of *Desmopsistrunciflora* (Schltdl. & Cham.) G.E. Schatz (Cuevas et al. 2002) and *Nectandrarudis* C.K. Allen (Cuevas et al. 2008), amongst others. Fortunately, the area forms part of the Sierra de Manantlán Biosphere Reserve, ensuring a certain degree of conservation of the flora of this area, which is notable in the region for its uniqueness and the distribution of some of its species (Cuevas 2002).

The species has been recorded in flower in the months of July-August and with ripe fruits from February to March.

##### Etymology.

The species honours the memory of Ramiro Cuevas Guzmán, dear brother of the first author, a person who loved the countryside and who collected plants for more than 30 years together with the author of the species, in various regions of the southern coast of Jalisco. Several of these collections are deposited in national herbaria and some have resulted in scientific novelties.

##### Preliminary conservation status.

According to the Categories and Criteria of the IUCN Red List (IUCN 2012), *L.ramiroi* is assigned a preliminary status of “Critically Endangered” CR B2a C2a(ii). The known and estimated geographical distribution of this species is less than 10 km^2^ in area and it has been recorded at only one locality, with a population size estimated at fewer than 250 mature individuals. Moreover, it should be noted that there is little natural regeneration of the species and few juvenile individuals present.

##### Additional specimens examined.

Mexico: State of Jalisco: Municipality of Casimiro Castillo: cañada El Tecolote, 19°36'55"N, 104°18'27"W, 13 December 1998 (st), *R. Cuevas, L. Guzmán* & *J. Aragón 6374* (ZEA); *R. Cuevas, L. Guzmán* & *J. Aragón 6367* (ZEA); cañada El Tecolote, 19°37'06"N, 104°19'29"W, 14 December 1998 (st), *R. Cuevas, L. Guzmán & J. Aragón 6413* (ZEA); cañada de La Naranjera, 19°37'20"N, 104°20'38"W, 23 February 1999 (ripe fr), *R. Cuevas, L. Guzmán* & *J. Aragón 6571* (ZEA); cañada de La Naranjera, 19°37'21"N, 104°20'33"W, 24 February 1999 (ripe fr), *R. Cuevas, L. Guzmán* & *J. Aragón 6591* (ZEA); barranca de La Naranjera, 19°36'55"N, 104°18'27"W, 11 July 1999 (immature fr), *R. Cuevas, L. Guzmán, C. Palomera* & *J. Aragón 6819* (ZEA); 19°36'55"N, 104°18'27"W, 11 July 1999 (fl), *R. Cuevas, L. Guzmán*, *C. Palomera* & *J. Aragón 6820* (ZEA).

##### Notes.

Due to its erect tepals, the exserted stamens with their filaments fused at the base, the anthers with large locules orientated in the longitudinal axis of the flower, with the valves opening upwards, *Licariaramiroi* is classified in the subgen.Licaria, within the group of species with exserted stamens and with the locules on the external surface of the anthers (Kurz 2000). Morphological characters with narrow leaves with 12 or more secondary veins, the shape and size of the flowers, exserted stamens and the large sporangia covering most of the anther body and running lengthwise to the flower axis, relate *Licariaramiroi* to L. *siphonantha* Lorea-Hern. but separates from this species by its fused tepals at its base, generally shorter and glabrous inflorescences, free anthers and the hypanthium internally sericeous (see Table 1). The shape and size of the flowers, the tepals fused at their bases, the glands often fused at their bases with free apices, the size of the cupules in the fruit with strongly undulating margins, as well as the shape and size of the fruit, relate *L.ramiroi* with the variable *L.triandra* (sensu Kurz (2000), including *L.cervantesii* (Kunth) Kosterm. and *Misantecanayaritensis* Lundell), the only species of *Licaria* recorded for western Mexico (Kurz 2000), from which it is separated by the presence of stamens fused only in the filaments, while the anthers are generally free, an internally sericeous hypanthium and other characters that are presented in Table 1.

**Figure 2.**
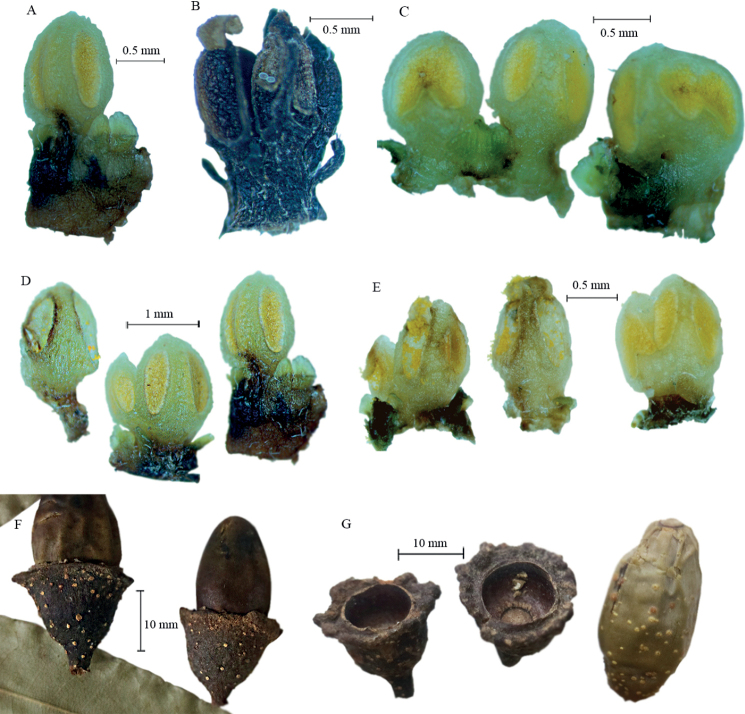
*Licariaramiroi* Cuevas sp. nov. **A** stamen with two pairs of glands **B** ring of stamens, with the anthers showing locules and valves and three glands in the base **C** three stamens of a flower, one showing a sagittate locule (left), the other with an irregular locule (right) and several glands **D** four stamens of a flower with several glands **E** five stamens of a flower **F** fruits with their cupules **G** one fruit and two cupules, showing the outer and inner margins.

A review of 60 flowers from two individuals (20 from one and 40 from the other) collected 23 years apart, suggests that the proportion of flowers with 3, 4 and 5 stamens in whorl III is more or less equal. This phenomenon observed in *L.ramiroi* is something previously not recorded in the genus *Licaria*. The evidence supporting the notion that they are all stamens of whorl III is that all of the anthers are extrorse and there is incomplete separation of stamen primordia and, when this occurs, some of these stamens can show a certain degree of fusion at the base of the anthers (see Fig. 2). Gradation is also observed in the locules that show an incomplete separation during development, sagittate locules and others with an irregular shape. Between four and eight glands were observed in *L.ramiroi*, some of them free and others fused at their bases, but always with clearly separated apices (Fig. 2). In *L.ramiroi*, the development of more than three fertile stamens in whorl III, with the corresponding increase in glands, could be a teratological condition due to an environmental response, possibly to the infection of Diptera larvae, which were observed parasitising some of the dissected flowers. To determine if the aberrations observed in the flowers of the species are due to the presence of the indicated parasites, further research will be required.

*Licaria* has been a well-defined genus by its three bisporangiate fertile stamens in whorl III, the presence or absence of staminodes in whorls I and II and the lack of staminodes in whorl IV, a deep receptacle and a double-margined cupule (Kurz 2000). Recent molecular studies have recovered a clade within the *Ocotea* complex called “*Licaria* group and allies”, which includes, in addition to *Licaria*, other genera with deep receptacles and a double-margined cupule, including species of *Ocotea* Aubl., *Aniba* Aubl., *Dicypellium* Nees & Mart., *Kubitzkia* van der Werff, *Paraia* Rohwer, H.G. Richt. & van der Werff and *Urbanodendron* Mez (Trofimov et al. 2019).

The double-margined cupule in *L.ramiroi* suggests a morphological connection to species in genera such as *Aiouea* Aubl., *Damburneya* Raf. and *Mespilodaphne* Nees & Mart., especially with those with bisporangiate anthers. However, the species of these genera present other characteristics that are not recorded in *L.ramiroi*, such as the fact that, in *Aiouea*, there are staminodes in the fourth androecial whorl that show a large glandular head, generally chordate to sagittate (Rohde et al. 2017); in *Damburneya*, the tepals are adaxially pubescent at the base and generally papillose towards the tips (Trofimov et al. 2016) and both genera have nine fertile stamens, occasionally six or three in *Aiouea*. *Mespilodaphne*, on the other hand, is a recently reinstated genus, it presents flowers with spreading tepals, tongue-shaped stamens, heavily papillose and tetrasporangiate (Trofimov et al. 2019). *Mocinnodaphne* Lorea-Hern., recently included in *Aiouea*, meets most of the characters of *Licaria*, with the exception of having well-developed staminodes in whorl IV (Lorea-Hernández 1995). Due to a set of characters present in *L.ramiroi*, such as its deep receptacle, fertile stamens in the III whorl, bisporangiate, cupule with double margin, lack of staminodes in the first two whorls and the fourth, we consider that the best inclusion of the species is in *Licaria*.

Understanding the morphological complexity of the “*Licaria* group and allies” within the *Ocotea* complex and taking into account the androecial morphology recorded in *L.ramiroi*, considering that it is a genetic condition, then the species could be relocated to another genus within the *Ocotea* complex, but it would be necessary to wait for the species to be included in some molecular study to better define its phylogenetic relationships.

## Supplementary Material

XML Treatment for Licaria ramiroi

## References

[B1] Burger WC van der Werff H (1990) Family #80 Lauraceae. In: Burger WC (Ed.) Flora Costaricensis. Fieldiana, Botany, new series 23: 1‒138. 10.5962/bhl.title.2586

[B2] Cuevas GR (2002) Análisis de gradientes de la vegetación de la cañada El Tecolote, en la sierra de Manantlán, Jalisco, México (Tesis doctoral). Colegio de Posgraduados, Montecillo, Texcoco, México.

[B3] Cuevas GR Cochrane TS (1999) *Beilschmiediamanantlanensis* (Lauraceae), una nueva especie de Jalisco, México. Novon 9(1): 18–21. 10.2307/3392109

[B4] Cuevas GR Vázquez-García JA (2021) Nueva especies de *Sideroxylon* de pétalos indivisos (Sapotaceae) para Jalisco, México. Revista Mexicana de Biodiversidad 92(0): e923535. 10.22201/ib.20078706e.2021.92.3535

[B5] Cuevas GR López-Mata L García-Moya E (2002) Primer registro de *Desmopsistrunciflora* (Schlecht. & Cham) G.E. Schatz (Annonaceae) para el occidente de México y análisis de su población en la sierra de Manantlán, Jalisco. Acta Botánica Mexicana 58(58): 7–18. 10.21829/abm58.2002.887

[B6] Cuevas GR García-Moya E Vázquez-García JA Núñez-López NM (2008) Estructura poblacional y relaciones ambientales del árbol tropical *Nectandrarudis* (Lauraceae), una especie rara en el occidente de México. Revista de Biología Tropical 56: 247–256. 10.15517/rbt.v56i1.5521 18624240

[B7] Gómez-Laurito J Cascante A (1999) *Licariacaribaea* (Lauraceae); A new species from the Carribean Lowlands of Costa Rica. Novon 9(2): 199–201. 10.2307/3391798

[B8] IUCN (2012) IUCN red list categories and criteria, version 3.1. 2^nd^ edn. IUCN, Gland, Switzerland and Cambridge U.K., [iv +] 32 pp. https://portals.iucn.org/library/node/10315

[B9] JSTOR (2020) Global Plants. ITHAKA, New York. http://plants.jstor.org

[B10] Kurz H (2000) Revision der Gattung *Licaria* (Lauraceae). Mitteilungen aus dem Institut für allgemeine Botanik in Hamburg 28/29: 89‒221.

[B11] Lorea-Hernández FG (1995) *Mocinnodaphne*, un género nuevo de la familia Lauraceae en la flora de México. Acta Botánica Mexicana 32(32): 25–32. 10.21829/abm32.1995.743

[B12] Lorea-Hernández FG (1999) Una nueva especie de *Licaria* (Lauraceae) del sur de México. Polibotánica 10: 105–110. http://www.redalyc.org/articulo.oa?id=62101006

[B13] Lorea-Hernández FG (2002) La familia Lauraceae en el sur de México: Diversidad, distribución y estado de conservación. Botanical Sciences 71(71): 59–70. 10.17129/botsci.1663

[B14] Lorea-Hernández FG (2005) Nuevas especies de *Licaria*, *Ocotea* y *Persea* (Lauraceae) de México. Acta Botánica Mexicana 71(71): 61–87. 10.21829/abm71.2005.995

[B15] Radford AE Dickison WC Massey JR Bell CR (1974) Vascular plant systematics. Harper and Row Publishers. Nueva York, U.S.A.

[B16] Rohde R Rudolph B Ruthe K Lorea-Hernández FG Rodríguez de Moraes PL Li J Rohwer JG (2017) Neither *Phoebe* nor *Cinnamomum* – the tetrasporangiate species of *Aiouea* (Lauraceae). Taxon 66(5): 1085–1111. 10.12705/665.6

[B17] Trofimov D Rudolph B Rohwer JG (2016) Phylogenetic study of the genus *Nectandra* (Lauraceae), and reinstatement of *Damburneya*. Taxon 65(5): 980–996. 10.12705/655.3

[B18] Trofimov D Rodríguez de Moraes PL Rohwer JG (2019) Towards a phylogenetic classification of the *Ocotea* complex (Lauraceae): Classification principles and reinstatement of *Mespilodaphne*. Botanical Journal of the Linnean Society 190(1): 25–50. 10.1093/botlinnean/boz010

[B19] Tropicos (2022) Tropicos.org. Missouri Botanical Garden. http://www.tropicos.org

[B20] van der Werff H (1991) A key to the genera of Lauraceae in the New World. Annals of the Missouri Botanical Garden 78(2): 377–387. 10.2307/2399567

[B21] van der Werff H (2009a) Nine new species of *Licaria* (Lauraceae) from tropical America. Harvard Papers in Botany 14(2): 145–159. 10.3100/025.014.0206

[B22] van der Werff H (2009b) Lauraceae (in part). Flora Mesoamericana 2(1): 1‒248. http://legacy.tropicos.org/Name/42000016?projectid=3&langid=66

[B23] van der Werff H Richter HG (1996) Toward an improved classification of Lauraceae. Annals of the Missouri Botanical Garden 83(3): 409–418. 10.2307/2399870

[B24] van der Werff H Vicentini A (2000) New species of Lauraceae from Central Amazonia, Brazil. Novon 10(3): 264–297. 10.2307/3393111

